# Parallelized SLAM: Enhancing Mapping and Localization Through Concurrent Processing

**DOI:** 10.3390/s25020365

**Published:** 2025-01-09

**Authors:** Francisco J. Romero-Ramirez, Miguel Cazorla, Manuel J. Marín-Jiménez, Rafael Medina-Carnicer, Rafael Muñoz-Salinas

**Affiliations:** 1 Departamento de Teoría de la Señal y Comunicaciones y Sistemas Telemáticos y Computación, Campus de Fuenlabrada, Universidad Rey Juan Carlos, 28942 Fuenlabrada, Spain; francisco.romero@urjc.es; 2 Departamento de Ciencia de la Computación e Inteligencia Artificial, Carretera San Vicente del Raspeig s/n, Universidad de Alicante, 03690 San Vicente del Raspeig, Spain; miguel.cazorla@ua.es; 3 Departamento de Informática y Análisis Numérico, Edificio Einstein, Campus de Rabanales, Universidad de Córdoba, 14071 Córdoba, Spain; mjmarin@uco.es (M.J.M.-J.); rmedina@uco.es (R.M.-C.); 4 Instituto Maimónides de Investigación en Biomedicina (IMIBIC), Avenida Menéndez Pidal s/n, 14004 Córdoba, Spain

**Keywords:** SLAM, lifelong mapping, localization, offline processing, parallel mapping

## Abstract

Simultaneous Localization and Mapping (SLAM) systems face high computational demands, hindering their real-time implementation on low-end computers. An approach to addressing this challenge involves offline processing, i.e., a map of the environment map is created offline on a powerful computer and then passed to a low-end computer, which uses it for navigation, which involves fewer resources. However, even creating the map on a powerful computer is slow since SLAM is designed as a sequential process. This work proposes a parallel mapping method pSLAM for speeding up the offline creation of maps. In pSLAM, a video sequence is partitioned into multiple subsequences, with each processed independently, creating individual submaps. These submaps are subsequently merged to create a unified global map of the environment. Our experiments across a diverse range of scenarios demonstrate an increase in the processing speed of up to 6 times compared to that of the sequential approach while maintaining the same level of robustness. Furthermore, we conducted comparative analyses against state-of-the-art SLAM methods, namely UcoSLAM, OpenVSLAM, and ORB-SLAM3, with our method outperforming these across all of the scenarios evaluated.

## 1. Introduction

Simultaneous Localization and Mapping (SLAM) systems represent advanced technology that has significantly transformed how we interact with our environment and opened new frontiers in fields such as robotics and computer vision [1,2,3].

SLAM techniques involve complex tasks such as environment perception, key feature identification and matching, motion tracking, position and orientation estimation, and constructing and updating a detailed environment map [4,5]. The interaction of all of these tasks demands substantial computing power and precise synchronization to achieve effective navigation. Furthermore, SLAM tasks become challenging when systems operate in open environments and must explore large spaces. As the camera moves and the mapped area grows, the system’s complexity increases, increasing the time required for optimization.

SLAM systems are widely used by mobile robots and embedded devices, which have severe hardware limitations. Thus, a robotic system capable of performing SLAM requires a powerful onboard processing unit, limiting its mobility, primarily due to its weight and electrical demand [6].

Reductions in hardware costs have significantly increased personal computers’ processing capacity. These advances include improvements in the processing algorithms, resulting in more powerful hardware and efficient optimization techniques. This has allowed SLAM systems to parallelize core tasks across multiple threads, such as tracking and mapping [1,2,7]. However, current SLAM systems are still primarily sequential processes in which one frame is processed after another. The recent work ReSLAM [8] proposed a method for reusing maps across multiple cameras by sequentially processing a map offline. Once the map is created, it can be transferred to the robots, allowing them to navigate the environment without the added concern of real-time control.

This work introduces a novel offline mapping approach coined as pSLAM (Parallel Simultaneous Localization and Mapping). The main objective of pSLAM is to harness and optimize the computational potential inherent in current computer systems to enhance the performance of visual SLAM systems. Unlike SLAM systems that process a video sequence frame by frame, beginning with the first frame and concluding with the last, our method advocates for partitioning the video sequence into multiple segments, corresponding to the number of available processing units at any given moment. Each segment is employed to create a submap that is later merged with the rest, yielding a consolidated representation of the explored environment. Please note that this work does not propose a SLAM method but a way to parallelize the mapping part in large scenarios.

Our work is similar to the Structure from Motion (SfM) problem; both create an offline environment map. However, while SfM excels at obtaining a three-dimensional point cloud from a set of manually selected images, in our case, the input is a long video sequence that cannot be fed directly to an SfM engine; it is necessary to select a set of candidates automatically. Another difference is that SLAM can take advantage of the spatio-temporal vicinity of the frames, which general SfM engines do not assume. Consequently, SLAM methods can operate better in visually repetitive environments such as buildings. Finally, let us stress that this work does not aim to propose a new SLAM method but a parallelization strategy for the map building process of existing SLAM methods. Consequently, the maps created with our method can be directly employed for robot navigation.

The rest of this paper is structured as follows. Section 2 reviews the works most closely related to this one, while Section 3 introduces the elements of SLAM and their mathematical formulation. Section 4 explains our method, and Section 5 presents the obtained results. Finally, Section 6 concludes and outlines future directions.

## 2. Related Works

The development of real-time algorithms for mobile system localization in an unknown environment has been a significant challenge since its inception. The work by Eade and Drummond [9,10] and Davison et al. [11] marked a starting point for visual Simultaneous Localization and Mapping (vSLAM) systems, achieving real-time and drift-free performance. In both systems, mapping and tracking are closely intertwined, with camera pose estimation and updates to the information on the three-dimensional environment co-occurring. An approach to improving the performance was the PTAM work [7], which proposed separating the mapping and tracking tasks into two different threads. Adopting a keyframe-based approach, which exclusively integrates relevant frames into the mapping process, enabled the substitution of filtering techniques with the application of global optimization methods, such as bundle adjustment. Despite being computationally costly, these methods provide much more accurate results. Following this line of thought, Mur-Artal et al. introduced ORB-SLAM [12], a monocular SLAM system based on keyframes that use ORB [13] features for tracking, mapping, relocalization, and loop-closing, employing a bag-of-words (BoW) method [14]. Subsequently, this work was extended with ORB-SLAM2 [15], which works with monocular, stereo, and RGB-D cameras. Later on, OpenVSLAM [16] offered a versatile vSLAM framework that is easy to customize to suit the user requirements while also being capable of working with various camera models, such as perspective, fisheye, and equirectangular models. Recently, the ReSLAM [8] work introduced a novel methodology for reusing the maps created during the SLAM process. While traditional methods generate maps tightly coupled to the characteristics of the cameras used for their construction (i.e., the image resolution and intrinsic parameters), ReSLAM proposes a new bottom-up pyramidal image representation, enabling the map to be reused by sensors with entirely different configurations. Finally, within keyframe-based systems, the UcoSLAM system [1] proposes a combination of natural and artificial markers [17], harnessing the advantages offered by both approaches. Despite the high performance exhibited by natural marker-based systems, they have limitations in repetitive and low-textured environments. In contrast to keypoints, these artificial markers demonstrate exceptional temporal stability, which aids in avoiding false relocalizations and loop closures.

On the other hand, a significant challenge that vSLAM systems must address is adapting to working in large and unknown environments. These systems construct and maintain a map of the explored environment to make this possible. This map serves the dual purpose of determining their positions and rectifying trajectory errors. In addition, real-time vSLAM systems require that the processing of the acquired data outpaces the rate at which new data are generated. This problem becomes more pronounced as the size of the map grows. To address large-scale and long-term problems, the authors of RTAB-Map [18] propose an online loop closure detection method based on memory management. The system maintains a limited graph of observations in working memory, while the rest are transferred into long-term memory. The same authors extended this concept [19], suggesting graph-based SLAM where multiple maps of the environment can coexist simultaneously in the system, solving the kidnapped robot problem and the initial state problem. Loop closure detection is employed throughout mapping sessions to verify whether the agent’s position coincides with a previous map. When such an occurrence arises, the system merges the maps, establishing a shared reference point and rectifying trajectory errors.

Castle et al. [20] introduced one of the earliest keyframe-based multi-map systems. This system can work with one or multiple cameras to construct individual maps. However, the system cannot merge or establish connections between distinct submaps. This issue was addressed in the work of [21], where the system could estimate the transformation between the two maps. Recently, Campos et al. [2] introduced ORB-SLAM3, a system based on a multi-map representation of the environment. When the system becomes lost, it initiates the creation of a new map, which is later merged with previous maps, contributing to the robustness of the mapping process.

Multiple maps have been widely employed in collaborative schemes, where multiple agents act as a distributed mapping system, with a central station responsible for processing the received information [22,23]. In the work proposed by Riazuelo et al. [24], C²TAM was introduced, a distributed framework where a group of agents is equipped with lightweight tracking systems capable of estimating their positions in the environment. These agents communicate with a central service responsible for handling the bulk of the storage and optimization operations. In CCM-SLAM [25,26], each agent works as a self-contained mapping and localization unit, capable of autonomously building and maintaining its local map. Simultaneously, these agents exchange information bidirectionally, facilitating the creation of a global map that collectively represents the entire environment explored. Cloud-based robotics applications are relatively new and hold significant potential, particularly in reducing the computational demands within autonomous vehicles quickly [27].

Two key aspects of the strategy followed during the map merging process are map alignment and data association [28]. On the one hand, map alignment involves transforming or rotating individual maps to ensure their proper alignment, ensuring that features and entities match coherently across maps. On the other hand, it is essential to establish mechanisms for feature or entity matching between maps so that correspondence between elements from different data sources is correctly identified [14,29]

Among the systems that handle multiple maps, AutoMerge [30] proposes a map alignment approach based on feature extraction using spherical harmonics, enabling the recognition of viewpoint-invariant places. Additionally, it employs an adaptive sequential alignment method for loop closure detection. MUI-TARE [31], which extends AutoMerge, introduces an adaptive approach to map merging, based on evaluating the quality of the feature association. On the other hand, S. Sunil et al. [32] present a map fusion methodology that incorporates spatial occupancy probability processing, along with feature detection using locally adaptive nonlinear diffusion filtering (KAZE features).

Recent advancements in SLAM methodologies have sought to address the challenges of computational efficiency, consistency, and adaptability in dynamic environments. The Markov Parallel Tracking and Mapping (MPTAM) framework [33] integrates parallel pipelines with the Markov property to ensure consistency in probabilistic estimation. Subsequently, the authors proposed the work [34], which focused on efficient real-time visual–inertial SLAM by asynchronously decoupling the front-end odometry and back-end optimization, ensuring consistency through global loop closure adjustments.

SG-SLAM [35] extends traditional SLAM systems by addressing the limitations of the static scene assumption, which often hinders the performance in dynamic environments. The framework incorporates two additional parallel threads: a semantic object detection thread to extract 2D semantic information and a semantic mapping thread to create 3D semantic objects and metric maps. RDS-SLAM [36] builds on ORB-SLAM3 by introducing parallel semantic and optimization threads for real-time performance. Unlike the traditional models, which first segment objects and then remove outliers from tracking, RDS-SLAM avoids delays by parallelizing semantic segmentation and using moving probabilities to exclude dynamic objects during tracking and mapping.

In this work, we introduce pSLAM, a novel system designed to maximize the speed of vSLAM processing. The proposed system takes as its input a video sequence of an environment and parallelizes its processing based on the computational resources available at that moment, resulting in a three-dimensional map of the environment. Our system was developed using our previous works on UcoSLAM [1] and ReSLAM [8] as the base layers. However, the proposed approach can be implemented on any other vSLAM system.

## 3. Method Overview

In this section, we will describe the key components of our SLAM system and the mathematical notation employed in this study.

### 3.1. Frames

Using a camera with the intrinsic parameters (optical center, focal length, and distortions) γ, let us define F={f} as the set of frames, with each comprising the following tuple:

f={θ,I,K},

where θ∈SE(3) is the estimated pose of the camera when image *I* was captured. On the other hand, $I=\{\left\{I^{l}\right\}|l>=1,l\in N\}$ is the image pyramid constructed by scaling *I* using several values of α∈[1,∞), ($I^{1}=I$), and K is the set of keypoints detected at different levels of the image pyramid I, obtained by applying a keypoint detector and subsequently a feature extractor, such as ORB [13].

Note that we will use subscripts throughout this paper to refer to a set included in a tuple. For instance, $K_{f}$ is the set of keypoints of frame *f*. Let us define a keypoint k∈K as the tuple

k={l,q,d},

where *l* represents the level of the image pyramid I at which the keypoint was detected, and $q\in R^{2}$ are its pixel coordinates in the image $I^{l}$. Additionally, each keypoint contains a binary feature descriptor denoted by *d*, which is represented by the following vector:

$$d=\left(d^{1},\ldots ,d^{n}\right)|d^{i}\in \left\{0,1\right\}.$$

### 3.2. Map

A SLAM system includes the necessary mechanisms to maintain an updated environment map as the camera moves. The following components define a map.

M={F,P,G,D}.

F⊆F represents the subset of frames used to estimate the camera pose and map the environment, known as keyframes. The set P={p} describes the map points generated by triangulating keypoints belonging to multiple keyframes. G is an undirected graph that interconnects the keyframes, where each vertex of the graph is represented by a weight indicating the strength of the connection. The weight is calculated based on the number of shared map points between interconnected keyframes. Finally, D is a bag-of-words (BoW) formed by the descriptors of the keyframes and is used for recognition in tasks such as relocalization and loop closure.

On the other hand, let us define a map point as a tuple.

$$p=\{x,O,\lambda ,\hat{d}\},$$

where $x\in R^{3}$ are the three-dimensional coordinates of the point, represented in the global reference system (grs); note that a map point is obtained by triangulating from multiple observations, i.e., several keypoints *k* belonging to different keyframes in the map. We denote the set of observations of map point *p* as

$$O_{p}=\left\{\left\{k\right\}|k\in K_{f},f\in F\right\}$$

Additionally, a map point *p* exhibits a direction vector $\lambda_{p}$, an average of the direction vectors from the keyframes $f^{i}$ observing the map point. In this way,

(1) $$\lambda_{p}=\frac{\sum_{i}^{n}{\theta_{3}^{-1}}_{f^{i}}}{\left|\right|\sum_{i}^{n}{\theta_{3}^{-1}}_{f^{i}}{\left|\right|}_{2}^{2}},f^{i}\in F,$$

where ${\theta_{3}^{-1}}_{f^{i}}$ represents the vector corresponding to the third column of the inverse transformation matrix θ.

Lastly, we consider the descriptor $\hat{d}$ of the map point *p* as the keypoint descriptor that minimizes its Hamming distance *H* to the rest of the observations $O_{p}$.

$${\hat{d}}_{p}=\underset{k_{d}^{i},k_{d}^{j}\in O^{p}}{\mathrm{argmin}}\sum_{i\neq j}H\left(k_{d}^{i},k_{d}^{j}\right)$$

### 3.3. Camera Pose

Let us define the set of map points observed in frame *f* as

$$C^{f}=\left\{\left\{p,k\right\}|p\in P,k\in f_{K}\right\},$$

where *p* is a map point and *k* is a keypoint detected in the image pyramid $I_{f}$ that was used in the triangulation to obtain *p*.

The camera pose estimation in a frame $\theta_{f}$ is determined by an error minimization function in the projection of the set $C^{f}$ of map points observed by the frame.

Then, let us define the projection of a single map point $p\in R^{3}$ through the following function:

(2) T(θ,p,γ),

where θ is the transformation matrix that allows us to move *p* from the global reference system (grs) to the camera reference system (crs), and γ are the intrinsic parameters of the camera used to project *p* onto the image plane. Therefore, the projection error of a three-dimensional point *p* that we are observing in the image with coordinates $u\in R^{2}$ can be calculated using the following equation:

(3) e(θ,p,γ,u)=T(θ,p,γ)−u

Using the previous function, we obtain the projection error of the set of map points observed using frame *f* through the following equation:

(4) $$\zeta \left(C^{f}\right)=\sum_{\left(p,k\right)\in C^{f}}Qe\left(\theta_{f},p,\gamma ,k\right)\Omega e{\left(\theta_{f},p,\gamma ,k\right)}^{T},$$

where *Q* is the Huber loss function used to reduce the influence of outliers in the optimization process, and Ω is the matrix responsible for modulating the weight of the keypoints according to the level of the image pyramid in which each of them has been detected.

$$\Omega =\alpha^{k_{l}}I_{2\times 2},$$

where $I_{2\times 2}$ is the identity matrix.

In this way, the keypoints detected in higher levels of the pyramid will have more relevance than those detected in lower levels corresponding to lower-resolution images.

Finally, the estimation of the camera’s position in frame *f* is determined by the function that minimizes the projection error of the observed map points.

(5) $${\hat{\theta }}_{f}=\underset{\theta_{f}}{\mathrm{argmin}}\left(\zeta \left(C^{f}\right)\right).$$

## 4. The Proposed Method

This section describes the proposed method, which aims to process a given video sequence as the input and utilize the SLAM approach to generate a map of the environment.

Unlike current SLAM systems, where the sequence is processed sequentially from the first frame to the last, our system divides the sequence into *m* subsequences processed in parallel, generating *m* submaps of the environment. Afterward, these submaps are unified and optimized, resulting in a single, comprehensive final map.

**Figure 1:**
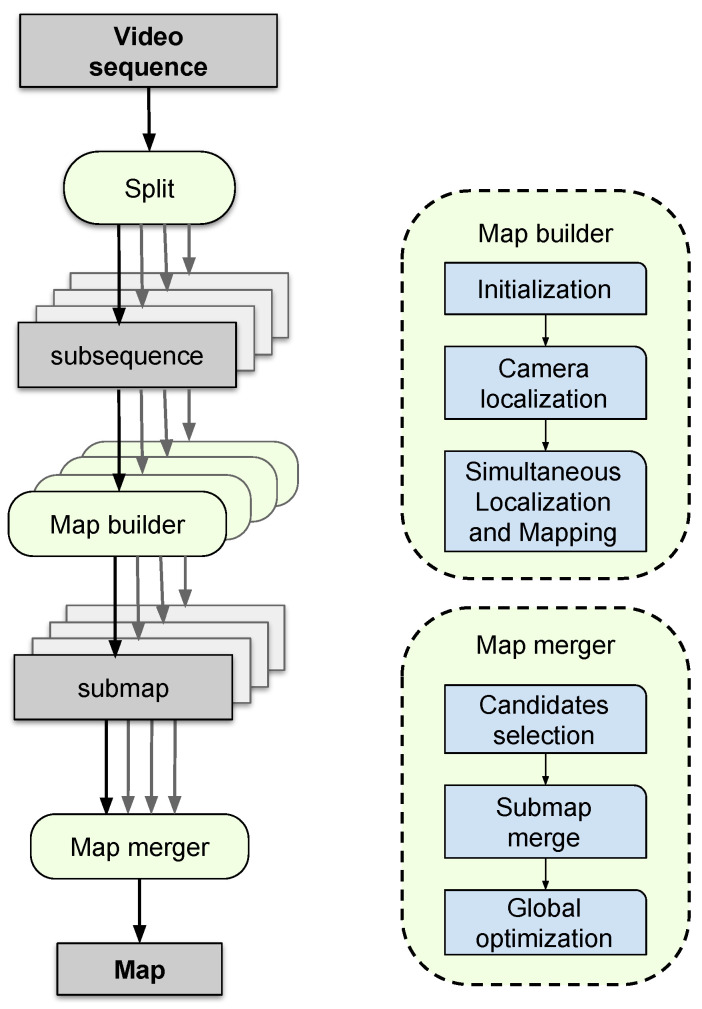
This image shows the main modules of our system. Initially, the video sequence is divided into multiple subsequences, with each processed by dedicated Map builder modules. Finally, each module’s resulting maps are merged to create a unified representation.

Figure 1 provides an overview of the system’s operation. First, the input video sequence is divided into *m* subsequences using the Split module (Section 4.1). Subsequently, each subsequence is processed by *m* SLAM processing modules, referred to as Map builders (Section 4.2). Finally, the previous phases generate *m* submaps that are merged by the Map merger module (Section 4.3).

### 4.1. Video Splitting

As an initial step, we start with a video sequence of the scene, denoted as *V*. To enable efficient and parallel processing, we partition *V* into *m* subsequences, represented as $V=\{\left\{V^{1},V^{2},\ldots ,V^{m}\right\}|m\geq 2\}$, according to the number *m* of available processing cores. Subsequently, these subsequences undergo concurrent processing in the next stage, outlined in Section 4.2. It is important to highlight that each subsequence consists of a subset of frames extracted from the original video sequence $V^{i}=\left\{\left\{f^{s_{i}},\ldots ,f^{s_{i}+\tau_{i}}\right\}|f^{j_{i}}\in F,\right\}$, where $f^{s_{i}}$ and $f^{s_{i}+\tau_{i}}$ represent the starting and ending frames, and $\tau_{i}$ the number of frames in the subsequence $V^{i}$.

To determine the cutting points within the video sequence (i.e., the starting frame $f^{s_{i}}$ and ending frame $f^{s_{i}+\tau_{i}}$ of each subsequence), the following constraints were taken into account:

1. The maximum number of subsequences, denoted as *m*, that can be subdivided from the original video sequence *V* will be determined by the number of modules available ( Map builder) in the system. Each module will be responsible for SLAM processing a single subsequence from the video.
2. To achieve proper merging of the submaps, an overlap of β frames is considered between consecutive subsequences.
3. Finally, to ensure robust initialization of the submap, the starting frame $f^{s_{i}}\in V_{i}$ of the subsequence must exhibit sufficient reliability. To do so, it is crucial to initialize the submap using the first $\tau_{i}$ frames from the subsequence $\left\{f^{s_{i}},\ldots ,f^{s_{i}+\tau_{i}}\right\}$. The cutting region of the sequence may coincide with frames in which there is no sensor movement or abrupt rotations. In cases where the submap cannot be properly initialized, the subsequence $V^{i}$ is discarded from the set *V*, and its frames $f\in V^{i}$ are integrated into the preceding subsequence $V^{i-1}$.

The selection of β is relevant since large values help to merge the submaps (i.e., more common keypoints are available between the submaps) at the cost of higher computing times. In this work, β is selected as 100, which, based on our experience, provides good results in the dataset tested. However, we are considering the possibility of exploring automatic ways to obtain this value in the future.

### 4.2. The Map Builders

Concurrent processing is applied to each subsequence $V_{i}\in V$ by the respective Map builder modules. The role of the Map builder module is to execute the SLAM process on the assigned subset of frames and generate a submap of the environment as the resulting output.

We employed the same methodology proposed in works [1,15] for the SLAM process.

#### 4.2.1. Initialization

Each submap $M^{i}$ is independently initialized using a keypoint-based approach, following the method proposed by OrbSLAM2 [15]. Firstly, the initialization method takes the first two frames of the subsequence $V^{i}$ ($f^{s_{i}}$ and $f^{s_{i}+1}$), from which keypoints descriptors are extracted. Subsequently, the descriptor matches between both frames are used to compute the homography and the essential matrix, which allows us to evaluate the error in the projection of the triangulated 3D points.

If the proposed solution is considered valid, the map is initialized, and the two keyframes and the 3D points associated with the keypoints are added to the map $M^{i}$. Otherwise, the system uses the first reference frame $f^{s_{i}}$ with subsequent frames ($f^{s_{i}+2}$, $f^{s_{i}+3}$, …). If after *n* attempts, the initialization is unsuccessful, the reference frame used $f^{s_{i}}$ is replaced by $f^{s_{i}+n}$, and the process starts again with subsequent frames ($f^{s_{i}+n+1}$, $f^{s_{i}+n+2}$, …).

Note that according to the third constraint proposed in Section 4.1, the initialization must be performed over the first $\tau_{i}$ frames; otherwise, the subsequence $V_{i}$ will be discarded, and its frames will be incorporated into the subsequence $V^{i-1}$.

#### 4.2.2. Camera Localization

If the system has been successfully initialized, the camera pose in the current frame $\theta_{f^{i}}$ is estimated using the previous camera pose estimation $\theta_{f^{i-1}}$ as a starting point. The camera pose estimation minimizes the projection error in the set of map points observed by $f^{i}$.

Firstly, let us define the reference frame ${\hat{f}}^{i}$ as the keyframe in the map that has the highest number of matches with the immediately preceding frame $f^{i-1}$, as it is likely that they will be observed in the current frame $f^{i}$. These matches will allow us to estimate the initial camera pose $\theta_{f^{i}}$.

Subsequently, the set of map point matches is filtered to obtain a refined camera pose of $f^{i}$. Firstly, map points *p* with an angle greater than α between their viewing angle $\lambda_{p}$ and the camera direction are discarded. Then, the set of map point matches is projected onto the image $I^{1}\in I_{f_{i}}$, and a search is performed for keypoints that have a smaller Hamming distance to the map point descriptor $\hat{d}$ within a search radius *r*. Finally, the set of keypoints and map point matches are used to obtain the final pose of the frame.

#### 4.2.3. Mapping

The system’s information is updated throughout the SLAM process to provide smooth and robust tracking. It is important to determine an appropriate strategy for inserting and removing keyframes as the environment is being explored.

First, let us establish the reference keyframe ${\hat{f}}^{i}$ as the map keyframe that exhibits the greatest number of matches with the analyzed frame preceding the current one, namely $f^{i-1}$. Following the methodology proposed by [1,15], a frame is added to the map if it has a valid pose, and the number of map points matched with the current frame is below a threshold percentage $\epsilon_{p}$ of the total number of detected map points in the reference keyframe ${\hat{f}}^{i}$.

When a new keyframe is added to the map, the system can add new map points and consolidate existing ones. For each keypoint in the new keyframe, correspondence with keypoints from neighboring keyframes of the reference keyframe is searched for using the known poses of the keyframes. Once a point is added, the points follow a survival policy based on the number of times the map point is visible with different keyframes. For a map point to be considered stable in the system, it must be visible in at least two-thirds of the subsequent frames until two new keyframes have been incorporated into the system. Afterward, the point becomes stable and will only cease to be stable if it is visible in fewer than three keyframes.

Similar to the strategy followed for keypoints, the same approach is applied to keyframes to prevent the number of keyframes from growing indefinitely. Thus, a keyframe may be removed from the system if at least $\epsilon_{k}\%$ of the keypoints correspond to map points observed by at least three keyframes.

### 4.3. Map Merging

Given two submaps $M^{i}$ and $M^{i+1}$, the Map merger module is responsible for merging them into a single map ${\hat{M}}^{i}=\Gamma \left(M^{i},M^{i+1}\right)$. The resulting map from the fusion process is a composition of the keyframes and map points belonging to both maps.

The process Γ of merging two maps is described through the following subsections. Firstly, candidate-shared points between the two maps are determined. Subsequently, map $M^{i+1}$ is transformed and aligned with respect to $M^{i}$. Finally, global optimization of the resulting map is performed to eliminate drift from the merging process.

**Figure 2:**
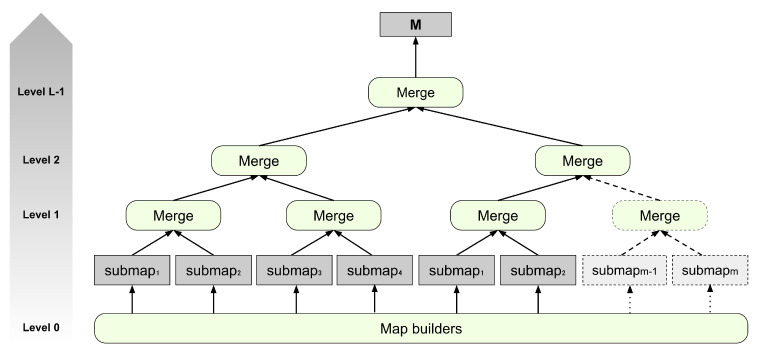
Representation of the methodology followed for merging the submaps. The merge modules are executed in parallel within the system, ensuring that each module can be executed only when the maps it will unify have previously been created.

Please note that the entire process of map merging is iterative and hierarchical, as shown in Figure 2. The inputs to the Merge modules at level 0 of the tree correspond to the maps generated by the Map builder modules. The input maps are generated as output from the immediately preceding level for the remaining levels of the tree. The process concludes when a single map is obtained, the final result of unifying the set of submaps provided as the input at level 0.

#### 4.3.1. Finding Merging Candidates

During the initial stage of the Zipper merge, the process involves searching for common map point observations between the two sets of keyframes (corresponding to $M^{i}$ and $M^{i+1}$ maps) to determine the merging regions. This is achieved by employing a BoW database approach $D_{M}$ (see Section 3.2), which enables the identification of similar keypoints based on their visual descriptors.

The procedure is iterative, as each keyframe $f\in F_{M^{i+1}}$ is queried against the database $D_{M^{i}}$ to retrieve a set of similar keyframes $f\in F_{M^{i}}$. Subsequently, each set of candidates is thoroughly examined, and only the most suitable one is considered a merging region, following a specific procedure.

To determine the most suitable candidate for each keyframe $f\in F_{M^{i+1}}$, their keypoints are matched with the keypoints of candidate keyframes. The three-dimensional positions of the observed map points are then utilized to estimate a rigid transformation using the RANSAC Perspective-n-Point method. A significant number of inliers indicates a reliable match, typically exceeding 30. Finally, among all the valid candidate keyframes, the one with the most matches is considered a valid link point for merging the maps.

#### 4.3.2. Initial Submap Merge

In the previous step, we identify the merging regions between the two maps. Once this is achieved, our next objective is to unify these regions into a single map. We achieve this by utilizing the map points that correspond to observations between pairs of keyframes (keypoint matches). These map points contain valuable 3D coordinates, which we leverage to find the optimal rigid transformation of $M^{i+1}$ using Horn’s method [37]. This transformation allows us to scale and align both maps within the same reference system.

Then, we keep the poses of the keyframes in the first map, $M^{i}$, fixed while optimizing the poses associated with map $M^{i+1}$ and its map points. This optimization process follows a similar procedure to the loop closure method proposed in [15]. By correcting the positions of the map points in $M^{i+1}$, we address any discrepancies and ensure accurate alignment with the first map.

(6) $$\underset{\theta_{f},f\in F_{M^{i+1}}}{\mathrm{argmin}}\zeta \left(C^{f}\right).$$

To streamline the map integration further, we merge duplicate map points that may exist within $M^{i+1}$. By eliminating redundancy, we enhance the map’s overall consistency and efficiency.

#### 4.3.3. Global Optimization

As a final step in merging two maps, global optimization $\hat{M}$ of the map *M* will be necessary to distribute the errors obtained during the merging process. The global optimization involves applying a transformation to the set of keyframe poses and map point positions, guided by an error minimization function using the Levenberg–Marquardt algorithm.

(7) $$\underset{\theta_{f}\in F_{M}}{\mathrm{argmin}}\zeta \left(C_{f}\right).$$

## 5. Experiments and Results

This section presents the experiments conducted to evaluate the performance of our pSLAM method. The source code and the data used in the experimentation are available for research purposes (https://www.uco.es/investiga/grupos/ava/portfolio/pslam, accessed on 5 January 2025). All experiments were conducted on a system equipped with an Intel(R) Xeon(R) CPU E5-2630 v4 @ 2.20GHz, with 46 GB of RAM (Intel Corporation, Santa Clara, CA, USA), using Ubuntu 20.04.2. We compared our method with state-of-the-art SLAM methods, namely UcoSLAM [1], OpenVSLAM [16], and ORBSLAM3 [2]. Table 1 shows the parameters defined in this work and the values used in the experiments.

UcoSLAM is designed to work in sequential and real-time modes. In real-time mode, frames are discarded to meet the real-time input flow of captured images, thus skipping frames from being added to the map if the map building thread is busy. The drawback of this approach is that we may obtain different results every time we run on the same video sequence. In sequential mode, however, the method does not skip any frame since the input image flow is stopped until the frame is fully processed, thus obtaining deterministic behavior. To properly compare the results, the sequential mode was employed. OpenVSLAM and ORBSLAM3, as provided by their authors, only allow for the real-time mode. Thus, to have a fair evaluation, we made the necessary modifications to their code to make them work sequentially. The modified versions of these systems are publicly available (https://www.uco.es/investiga/grupos/ava/portfolio/pslam, accessed on 5 January 2025).

A total of 42 video sequences were employed for the evaluation, with these belonging to three different datasets: Kitti [38] (Section 5.1), 4Seasons [39] (Section 5.2), and a dataset recorded by us (Section 5.3). Table 2 summarizes the video sequences used for each dataset.

To assess the performance of the different methods, we processed every sequence with each SLAM method, generating an environment map for each sequence. We then evaluated the average processing speed during the map construction (expressed in frames per second) and the absolute trajectory error (ATE), computed as the Root Mean Square Error between the ground truth and the estimated camera positions for each frame. It is worth noting that the ATE is only evaluated in frames where the system provides a valid position. The percentage of frames for which a valid position is obtained is called Trck and is also reported.

It is important to note that the UcoSLAM, ORBSLAM3, and OpenVSLAM methods start the map construction using the first frames in the sequence and end with the last frame of the video sequence. On the other hand, our method first divides the sequence into *m* subsequences, which are processed independently, resulting in *m* submaps. In the second phase, pairs of contiguous submaps are merged to finalize the map for each original video sequence. To evaluate our method, various configurations were considered, labeled as pSLAM-2, pSLAM-4, pSLAM-8, and pSLAM-16, meaning that each original video sequence was divided into m=[2,4,8,16] subsequences, respectively.

The rest of this section is structured as follows. First, Section 5.1 analyzes the system and compares it to state-of-the-art SLAM methods using the Kitti dataset [38]. Following this, the systems are analyzed in Section 5.2 using the 4Seasons dataset [39], and in Section 5.3, the CUR dataset is employed for the analysis [8]. Lastly, in Section 5.4, we analyze the computation times consumed by the different processes of our method.

### 5.1. The Kitti Dataset

The Kitti dataset [38] comprises a set of video sequences recorded in various residential areas, rural environments, and highways. The video sequences were captured using a stereo camera mounted on top of a car. The camera system is synchronized with a GPS and a laser scanner system, providing a precise ground truth for the trajectories. Many of the employed sequences depict challenging situations with multiple loops, rapid changes in direction, and lighting variations. The dataset consists of 20 video sequences (10 sequences from the left camera and 10 from the right camera), recorded at a resolution of 1240×376. Table 2 summarizes the sequences and the number of frames used in the experimentation.

Table 3 displays the average results on the ATE, Trck, and FPS for the pSLAM, OpenVSLAM, OrbSLAM3, and UcoSLAM methods using the Kitti dataset sequences. According to the obtained results, it can be observed that in general terms, our method exhibits a lower ATE than that of the other methods, achieving its best result when m=8. In terms of the percentage of tracked frames, all methods show values close to 100%. Lastly, our method demonstrates a better performance in terms of FPS, achieving a high performance when m=16.

**Figure 3:**
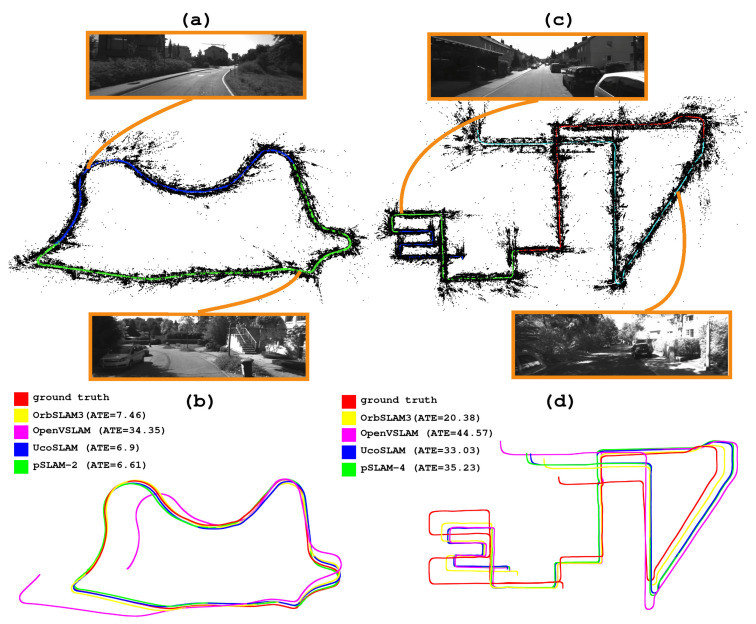
(**a**,**c**) Maps generated by our method, using two video sequences from the Kitti dataset. Different configurations of the parameter *m* were employed to create each map, m=2 and m=4, respectively. (**b**,**d**) Trajectories of the evaluated methods are depicted in various colors, along with the ground truth.

Figure 3a,c show the maps created by our method for two different sequences from the Kitti dataset. In Figure 3a, the resulting map is a product of merging two submaps, clearly distinguishable by the different colors assigned to their corresponding keyframes (blue and green). Similarly, the result of merging four submaps is shown in Figure 3c. Finally, Figure 3b,d show the trajectory followed by the different methods in these sequences, including the ground truth for comparison. As shown in Figure 3b, all of the methods follow the trajectory correctly (except for OpenVSLAM, where the loop closure fails). Conversely, Figure 3d reveals that although all methods follow similar trajectories, they do not align with and scale perfectly to the ground truth.

### 5.2. The 4Seasons Dataset

The 4Seasons dataset [39] comprises a collection of video sequences recorded in various scenarios, including industrial areas, highways, and local neighborhoods. Multiple sequences are provided for each scenario, including partially overlapping routes and multiple loops. The video sequences were recorded in different periods of the year, showcasing a wide range of changes in the lighting conditions, moving objects, and environmental conditions typical of the year’s four seasons. The recordings were made using a custom stereo–inertial sensor consisting of a pair of monochrome industrial-grade global shutter cameras (Basler acA2040-35gm) mounted onto a highly rigid aluminum rail with a stereo baseline of 30 cm. Each video sequence includes the ground truth for the entire frame trajectory.

Due to the large size of the dataset, we selected three different scenarios from the entire set of video sequences. For each scenario, we chose two recordings. Since each recording was made with a pair of monocular cameras, we used 12 video sequences recorded at a resolution of 800×400 for this experiment. Table 2 summarizes the set of video sequences and the number of frames used in the experimentation.

Table 4 presents the average results for the pSLAM, OpenVSLAM, ORB-SLAM3, and UcoSLAM methods using the 4Seasons sequences. According to the results, ORB-SLAM3 exhibits a better performance regarding the ATE and the percentage of correctly tracked frames. Please note that pSLAM is a parallelized version of UcoSLAM, and as such, we can not necessarily expect improvements in accuracy over it. However, regarding the FPS, ORB-SLAM3 performs more poorly than the other methods, with our method achieving the best results and the fastest processing times when m=16.

**Figure 4:**
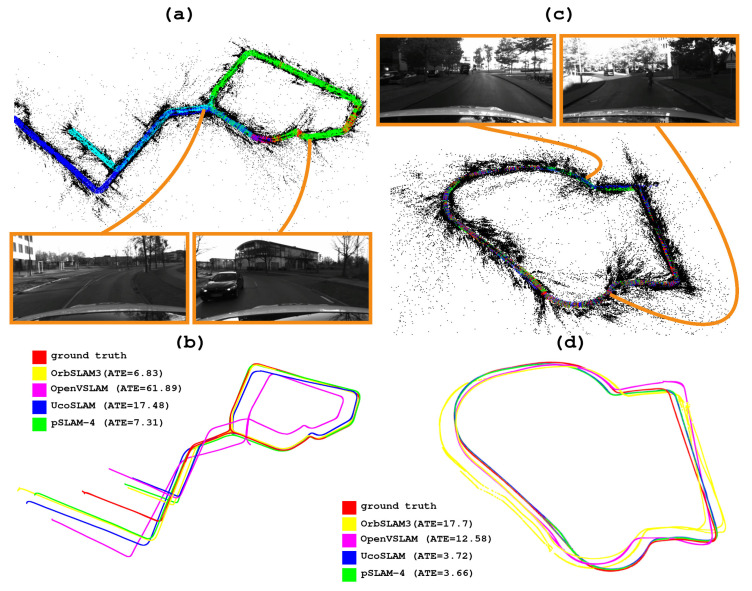
(**a**,**c**) Maps generated by our method for two video sequences of the 4Seasons dataset, using the parameter m=4. (**b**,**d**) Trajectories of the different methods in the same sequences.

Figure 4a,c show two maps created by our method for two different sequences. Both maps result from combining several maps, according to the *m* parameter (m=4 in both cases). Figure 4b,d compare the trajectory followed by our proposed method versus that of the state-of-the-art methods evaluated in the experiment.

### 5.3. The CUR Dataset

The Campus Universitario Rabanales (CUR) dataset collects 10 video sequences from five different outdoor scenarios at our university campus [8]. The dataset contents were recorded using two different monocular cameras with a resolution of 1920×1080 pixels and a frame rate of 30 fps. The first three scenarios (sequences 00, 01, and 02) were recorded at an average speed of 5 km/h by a person walking and holding the camera in his hands. For the last two scenarios, the sequences were recorded using a car at an average speed of 12 km/h. Table 2 summarizes the video sequences and the number of frames used in this dataset. For each sequence, a set of control points was collected along the trajectories to scale and align the estimated trajectory with the ground truth.

**Figure 5:**
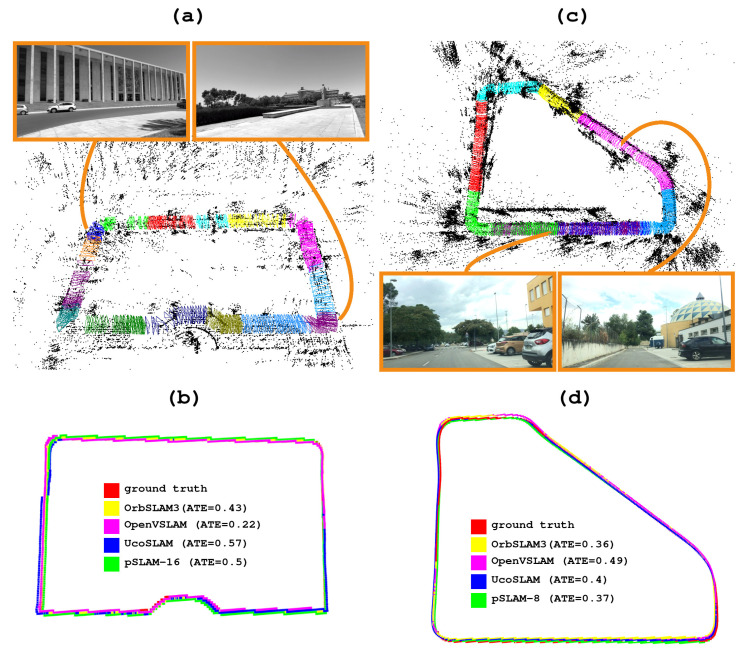
(**a**,**c**) Maps generated using two video sequences from the CUR dataset. Different parameter configurations *m* were employed to create the maps: (**a**) m=16, while (**c**) m=8. (**b**,**d**) Trajectories followed by the different methods evaluated.

Table 5 presents the results of the pSLAM, OpenVSLAM, ORB-SLAM3, and UcoSLAM methods for the CUR dataset. According to the results, we highlight the performance of our method for m=16, where our method outperforms the other methods in terms of the ATE, the percentage of frames correctly tracked, and the FPS. The ground truth trajectory derived from the control points and the different trajectories followed by our method and the state-of-the-art methods can be observed in Figure 5b,d.

Finally, Figure 5a,c show the maps created by our method for two different sequences of the CUR dataset. These maps result from merging *m* submaps, where Figure 5a corresponds to m=16 and Figure 5c corresponds to m=8. The trajectories followed by the ground truth, our method, and the state-of-art methods for comparison are shown in Figure 5c,d.

### 5.4. Computing Time Analysis

This section analyzes how the computing time of the different parts of our method varies as the number of partitions *m* changes, particularly for the values m=[2,4,6,8,16]. The use of the maximum value of 16 is motivated by the characteristics of the datasets used in the experiments, specifically the length of the sequences. For instance, in the dataset with the longest sequence, containing 17,764 frames, using this maximum value results in the creation of submaps comprising approximately 900 frames each, accounting for a 100-frame overlap between adjacent submaps. However, it is worth noting that this maximum value could be increased for even longer sequences if necessary.

We considered the key tasks that describe our approach, consisting of an initial phase in which the video sequence is divided into subsequences and the environment submaps are constructed (Section 4.1 and Section 4.2), which we label as map building. This is followed by a second phase of map merging (Section 4.3). The second phase encompasses the following processes: searching for the keyframes and candidate points of interest for merging ( Keyframe candidates), the initial merging of the two submaps into a single map ( Initial merge), and finally, global map optimization ( Global opt). Note that while the first phase, map building, is performed during the first level of processing, the remaining phases involved in the submap merging are employed for each level of the merging.

**Figure 6:**
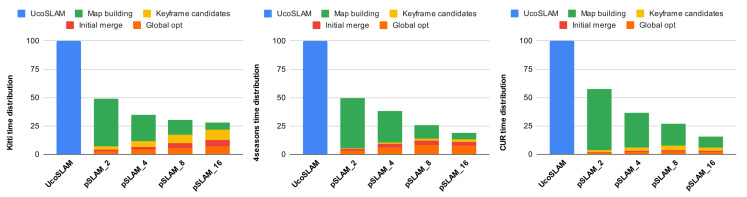
Computing time comparison of UcoSLAM and the proposed pSLAM method for different values of *m* on the datasets analyzed.

Figure 6 shows the computing time of UcoSLAM and our method for the sequences tested. The percentage of time consumed by different phases is displayed on the horizontal axis, with 100% representing the computing time of UcoSLAM. Please note that we are comparing against UcoSLAM since this is the SLAM system we parallelized with our approach. As can be observed, our method significantly reduces the computing time of the base algorithm UcoSLAM. While the maximum gain obtained in our tests is achieved using m=2, the improvement is clear across all configurations. The maximum time reduction is achieved in the CUR dataset, where pSLAM-16 consumes only 15.4% of the time used by UcoSLAM.

Furthermore, looking at the time distribution for each method, the time consumed by the map building phase decreases with the parameter *m* employed, mainly due to the parallelization of the submap construction. While for pSLAM-2, the map building process represents an average of 89.98% of the total time across the datasets, for pSLAM-16, it accounts for only 38.77% of the total processing time, allowing other processes such as Keyframe candidates and Global opt to have more weight in the total time. For the 4Seasons dataset, the Global opt phase increases in its weight relative to the other phases, rising from 6.26% for pSLAM-2 to 40% in pSLAM-16. This is due to the sequences’ complexity and length, where the video sequences’ division heavily influences the trajectory error correction process.

Finally, Table 6 provides a summary of the speedup of our method compared to UcoSLAM for the set of datasets used. The speedup is defined as the ratio of the time taken by our method to the time taken by the compared method. As shown, our method, pSLAM-2, is on average twice as fast as UcoSLAM, while pSLAM-16 is nearly six times faster.

## 6. Conclusions and Future Works

The high computational demands required by SLAM systems have been growing simultaneously with the size of the environments to be explored, making real-time SLAM on low-resource systems unfeasible. A widely adopted solution is offline processing of sequences on computers with a higher computing capacity, creating an environment map.

In this context, our work introduces pSLAM, a novel methodology for SLAM processing. Firstly, the system divides a video sequence into multiple subsequences according to the processing units available. Subsequently, each subsequence is processed independently, generating multiple submaps that are eventually merged into a single global map of the environment.

SLAM parallelization significantly enhances real-world applications like robotics and autonomous driving by accelerating mapping and localization. This ensures a faster, more accurate environmental understanding, enabling robots or vehicles to navigate complex, dynamic environments efficiently and safely. Nevertheless, our method, due to its offline processing, has inherent limitations that make it unsuitable for certain applications, such as autonomous exploration in unknown environments.

Experiments conducted in extensive scenarios demonstrate that our system significantly improves the computation times, and this improvement is directly correlated with the number of divisions performed while maintaining accuracy in tracking the trajectory and number of successful frames. Furthermore, we compared our system with various state-of-the-art SLAM methods, showcasing its superior performance in all of the evaluated scenarios, except for the 4Seasons dataset, where our method exhibits a poorer performance in terms of the ATE. This could be due to the complexity present in the dataset, where multiple loop closures occur for the same sequence.

One aspect not addressed in the current study is the impact of the characteristics of the selected overlap regions on the map quality. Specifically, overlap regions involving abrupt rotations may negatively influence the final results. As part of future work, we propose a comprehensive evaluation of this methodology’s performance under varying dynamic conditions, with a focus on variations in the camera’s speed and rotation. Moreover, it would be beneficial to develop a semi-automatic approach to this process, eliminating the need to predefine the number of frames used for overlapping.

To assess the proposed methodology, monocular sensor data were utilized. As an interesting avenue for future work, expanding this evaluation to incorporate other sensor types, such as RGBD, LiDAR, and stereo sensors, would provide a more comprehensive and robust analysis of the effectiveness of our approach.

## Figures and Tables

**Table 1:** Parameter values used by pSLAM in experiments.

Parameter	Value	Comment
$\tau_{i}$	50	Maximum number of frames used for initialization (Section 4.1)
β	100	Overlap of frames between consecutive subsequences (Section 4.1)
*m*	[2, 4, 8, 16]	Number of subsequences (Section 4.1)

**Table 2:** Information about the video sequences employed in our experiments.

Dataset	Sequence	Cameras	Frames
Kitti	00	cam0/cam1	4541/4541
	01	cam0/cam1	1101/1101
	02	cam0/cam1	4661/4661
	03	cam0/cam1	801/801
	04	cam0/cam1	271/271
	05	cam0/cam1	2761/2761
	06	cam0/cam1	1101/1101
	07	cam0/cam1	1101/1101
	08	cam0/cam1	4071/4071
	09	cam0/cam1	1591/1591
4Seasons	business_campus_1	cam0/cam1	10,720/10,720
	business_campus_2	cam0/cam1	12,013/12,013
	neighborhood_1	cam0/cam1	11,090/11,090
	neighborhood_2	cam0/cam1	10,020/10,020
	office_loop_1	cam0/cam1	14,960/14,960
	office_loop_2	cam0/cam1	12,583/12,583
CUR	00	cam0/cam1	4199/2262
	01	cam0/cam1	8938/6250
	02	cam0/cam1	17,764/8287
	03	cam0/cam1	4398/2930
	04	cam0/cam1	4475/3159

**Table 3:** The average results obtained using the video sequence from the Kitti dataset. For each method (pSLAM with m=2, m=4, m=8, m=16), OpenVSLAM, ORB-SLAM3, and UcoSLAM, the evaluation metrics include the absolute trajectory error (ATE), percentage of successfully tracked frames (Trck), and frames per second (FPS). Best results are marked in bold.

Method	ATE (m.)	Trck (%)	FPS
pSLAM-2	36.62	99.56	1.59
pSLAM-4	32.97	99.36	2.38
pSLAM-8	**24.04**	99.69	2.61
pSLAM-16	27.28	**99.77**	**2.73**
OpenVSLAM	60.30	99.05	1.49
ORB-SLAM3	47.85	**99.90**	0.53
UcoSLAM	42.13	99.56	0.69

**Table 4:** Average results obtained using the video sequence from the 4Seasons dataset. For each method (pSLAM with m=2, m=4, m=8, and m=16), OpenVSLAM, ORB-SLAM3, and UcoSLAM, the evaluation metrics include the absolute trajectory error (ATE), percentage of successfully tracked frames (Trck), and frames per second (FPS). Best results are marked in bold.

Method	ATE (m.)	Trck (%)	FPS
pSLAM-2	32.16	96.28	6.70
pSLAM-4	26.95	98.01	8.23
pSLAM-8	34.22	97.20	9.81
pSLAM-16	34.72	97.14	**12.33**
OpenVSLAM	48.24	97.70	5.83
ORB-SLAM3	**17.37**	**98.65**	0.86
UcoSLAM	36.35	92.35	2.25

**Table 5:** The average results obtained using the video sequence from the CUR dataset. For each method (pSLAM with m=2, m=4, m=8, and m=16), OpenVSLAM, ORB-SLAM3, and UcoSLAM, the evaluation metrics include the absolute trajectory error (ATE), percentage of successfully tracked frames (Trck), and frames per second (FPS). The best results are marked in bold.

Method	ATE (m.)	Trck (%)	FPS
pSLAM-2	0.89	99.81	17.14
pSLAM-4	1.75	99.82	27.45
pSLAM-8	1.44	99.79	37.08
pSLAM-16	**0.62**	**99.92**	**61.47**
OpenVSLAM	1.22	89.88	3.01
ORB-SLAM3	2.27	98.64	0.71
UcoSLAM	0.75	99.80	9.32

**Table 6:** Speedup of different configurations of pSLAM (pSLAM with m=2, m=4, m=8, and m=16) compared to UcoSLAM.

Dataset	pSLAM-2	pSLAM-4	pSLAM-8	pSLAM-16
Kitti	2.13	3.19	3.94	4.17
CUR	1.76	2.79	3.87	6.65
4Season	2.10	2.91	4.43	6.74
Avrg.	2.00	2.96	4.08	5.85

## Data Availability

The proposed methods and the employed data are available at https://www.uco.es/investiga/grupos/ava/portfolio/pslam (last accessed on 6 November 2024).
